# Supplementary material for: Severity of COVID-19 at elevated exposure to perfluorinated alkylates
Source: PLoS One. 2020 Dec 31;15(12):e0244815. doi: 10.1371/journal.pone.0244815 (PMC7774856; doi:10.1371/journal.pone.0244815)
Supplement: S1 Table — (DOCX) [file pone.0244815.s001.docx]

**S1 Table. Spearman’s correlation coefficients for pairwise comparisons of detectable PFASs in plasma from 323 subjects included in the study.**

|  | PFBA | PFBS | PFHXS | PFHpS | PFOA | PFOS | PFNA | PFDA |
| --- | --- | --- | --- | --- | --- | --- | --- | --- |
| PFBS | -0.0481 |  |  |  |  |  |  |  |
| PFHxS | 0.0520 | 0.0828 |  |  |  |  |  |  |
| PFHpS | 0.0491 | 0.0796 | 0.8916 |  |  |  |  |  |
| PFOA | 0.0617 | 0.1687 | 0.7072 | 0.7162 |  |  |  |  |
| PFOS | 0.0591 | 0.0936 | 0.8406 | 0.9375 | 0.7248 |  |  |  |
| PFNA | 0.0127 | 0.1395 | 0.7133 | 0.7698 | 0.7759 | 0.8406 |  |  |
| PFDA | 0.0141 | 0.1320 | 0.6102 | 0.6486 | 0.6050 | 0.7477 | 0.8930 |  |
| PFUdA | 0.0297 | 0.1920 | 0.5115 | 0.5286 | 0.5031 | 0.6279 | 0.8133 | 0.9150 |
