# Supplementary material for: Severity of COVID-19 at elevated exposure to perfluorinated alkylates
Source: PLoS One. 2020 Dec 31;15(12):e0244815. doi: 10.1371/journal.pone.0244815 (PMC7774856; doi:10.1371/journal.pone.0244815)
Supplement: S2 Table — (DOCX) [file pone.0244815.s002.docx]

**S2 Table. Median plasma concentrations of additional PFASs (25^th^-75^th^ percentiles) in ng/mL by population characteristics**

| **Population characteristics** | **No. of persons (%)** | **PFBS** | **PFHpS** | **PFDA** | **PFUdA** |
| --- | --- | --- | --- | --- | --- |
| **Total** | 323 (100) | 0.10 (<LOD, 0.18) | 0.12 (0.06, 0.19) | 0.14 (0.09, 0.22) | 0.09 (0.05, 0.18) |
| **Age (years)** |  |  |  |  |  |
| 30-39 | 37 (11) | 0.14 (0.08, 0.20) | 0.06 (0.04, 0.12) | 0.12 (0.09, 0.18) | 0.08 (0.05, 0.14) |
| 40-49 | 64 (20) | 0.13 (<LOD, 0.20) | 0.09 (0.05, 0.13) | 0.11 (0.08, 0.15) | 0.07 (0.04, 0.10) |
| 50-59 | 106 (33) | 0.11 (<LOD, 0.17) | 0.14 (0.06, 0.21) | 0.15 (0.10, 0.24) | 0.10 (0.05, 0.19) |
| 60-70 | 116 (36) | 0.04 (<LOD, 0.16) | 0.16 (0.09, 0.25) | 0.17 (0.12, 0.24) | 0.12 (0.06, 0.19) |
| P value ^a^ |  | 0.010 | <0.001 | <0.001 | 0.005 |
| **Sex** |  |  |  |  |  |
| Male | 174 (54) | 0.08 (<LOD, 0.18) | 0.16 (0.09, 0.24) | 0.14 (0.10, 0.24) | 0.09 (0.05, 0.17) |
| Female | 149 (46) | 0.11 (<LOD, 0.18) | 0.08 (0.04, 0.14) | 0.14 (0.09, 0.21) | 0.10 (0.05, 0.18) |
| P value ^b^ |  | 0.126 | <0.001 | 0.417 | 0.498 |
| **All chronic disease** |  |  |  |  |  |
| Yes | 222 (69) | 0.10 (<LOD, 0.17) | 0.11 (0.06, 0.18) | 0.14 (0.09, 0.21) | 0.09 (0.05, 0.16) |
| No | 101 (31) | 0.12 (<LOD, 0.20) | 0.12 (0.06, 0.20) | 0.15 (0.10, 0.25) | 0.11 (0.06, 0.21) |
| P value ^b^ |  | 0.104 | 0.950 | 0.109 | 0.040 |
| **Kidney disease** |  |  |  |  |  |
| yes | 34 (11) | 0.09 (<LOD, 0.17) | 0.15 (0.06, 0.20) | 0.16 (0.09, 0.25) | 0.11 (0.04, 0.18) |
| no | 289 (89) | 0.10 (<LOD, 0.18) | 0.11 (0.06, 0.18) | 0.14 (0.09, 0.22) | 0.09 (0.05, 0.18) |
| p-value ^b^ |  | 0.605 | 0.377 | 0.500 | 0.832 |
| **National origin** |  |  |  |  |  |
| Western Europe | 224 (69) | 0.11 (<LOD, 0.19) | 0.14 (0.08, 0.22) | 0.16 (0.11, 0.24) | 0.11 (0.06, 0.19) |
| Other | 99 (31) | 0.08 (<LOD, 0.17) | 0.07 (0.04, 0.14) | 0.10 (0.07, 0.16) | 0.05 (<LOD, 0.09) |
| P value ^b^ |  | 0.147 | <0.001 | <0.001 | <0.001 |
| **Place of inclusion** |  |  |  |  |  |
| Odense | 48 (15) | <LOD (<LOD, <LOD) | 0.12 (0.06, 0.19) | 0.14 (0.11, 0.20) | 0.07 (0.04, 0.13) |
| Copenhagen area | 275 (85) | 0.12 (0.03, 0.20) | 0.12 (0.06, 0.19) | 0.14 (0.09, 0.23) | 0.10 (0.05, 0.18) |
| P value ^b^ |  | <0.001 | 0.661 | 0.798 | 0.044 |
| **Timing of blood sampling** |  |  |  |  |  |
| After diagnosis - 1 week before | 193 (60) | <LOD (<LOD, 0.12) | 0.12 (0.06, 0.18) | 0.13 (0.09, 0.20) | 0.08 (0.04, 0.14) |
| >1 week - 1 year before | 46 (14) | 0.17 (0.12, 0.24) | 0.10 (0.05, 0.18) | 0.15 (0.10, 0.26) | 0.10 (0.05, 0.22) |
| > 1year before diagnosis | 84 (26) | 0.15 (0.11, 0.23) | 0.12 (0.07, 0.23) | 0.16 (0.10, 0.26) | 0.13 (0.07, 0.22) |
| p-value ^a^ |  | <0.001 | 0.408 | 0.012 | <0.001 |

^a^ Variables with more than two categories were tested using Kruskal-Wallis rank test
^b^ Binary variables were tested using Wilcoxon rank-sum test
