# Supplementary material for: Severity of COVID-19 at elevated exposure to perfluorinated alkylates
Source: PLoS One. 2020 Dec 31;15(12):e0244815. doi: 10.1371/journal.pone.0244815 (PMC7774856; doi:10.1371/journal.pone.0244815)
Supplement: S3 Table — (DOCX) [file pone.0244815.s003.docx]

**S3 Table. Ordered logistic regression odds ratios (ORs) of increased COVID-19 severity at an increase by 1 ng/mL in plasma concentrations of additional PFASs**

| **PFAS** | **No. of persons** | **OR (95% CI)** | | **No. of persons** | **OR (95% CI)** |
| --- | --- | --- | --- | --- | --- |
|  |  | **Crude** | **Adjusted for main covariates^a^** |  | **Exposure at time of diagnosis^a,b^** |
| PFBS (>LOD/<LOD) ^d^ | 207/68 | 0.28 (0.17, 0.47) | 0.65 (0.36, 1.19) | 62/60 | 1.52 (0.67, 3.47) |
| PFHpS (ng/mL) | 323 | 1.19 (0.22, 6.40) | 0.17 (0.02, 1.43) | 170 | 0.03 (0.00, 1.24) |
| PFDA (ng/mL) | 323 | 0.54 (0.12, 2.48) | 0.53 (0.10, 2.84) | 170 | 0.46 (0.04, 5.76) |
| PFUdA (ng/mL) | 323 | 0.11 (0.02, 0.83) | 0.32 (0.03, 3.08) | 170 | 0.15 (0.00, 7.29) |

^a^ Adjusted for age, sex, kidney disease, other chronic disease, national origin, place of testing, and days between blood sampling and diagnosis
^b^ Excluding individuals who had blood sampled more than one week before or after diagnosis
^c^ PFBS analyses were conducted for samples from Copenhagen area only
