# Supplementary material for: Severity of COVID-19 at elevated exposure to perfluorinated alkylates
Source: PLoS One. 2020 Dec 31;15(12):e0244815. doi: 10.1371/journal.pone.0244815 (PMC7774856; doi:10.1371/journal.pone.0244815)
Supplement: S4 Table — (DOCX) [file pone.0244815.s004.docx]

**S4 Table. Logistic regression odds ratios (ORs) of increased COVID-19 severity at an increase by 1 ng/mL in plasma concentrations of all PFASs detected**

| **PFAS** | | | **Hospitalization v. no hospitalization** | | **Intensive care unit and/or deceased v. hospitalization** | |
| --- | --- | --- | --- | --- | --- | --- |
|  |  |  | **No. of persons** | **Adjusted OR (95% CI) ^a^** | **No. of persons** | **Adjusted OR (95% CI) ^a^** |
| PFBA | <LOD | | 108/84 | 1.89 (0.41, 8.73) | 27/108 | 5.18 (1.29, 20.72) |
|  | >LOD | | 54/24 |  | 26/54 |  |
| PFBS ^b^ | <LOD | | 50/5 | 0.22 (0.05, 0.93) | 13/50 | 2.33 (0.61, 8.87) |
|  | >LOD | | 78/99 |  | 30/78 |  |
| PFHxS | |  | 162/108 | 0.49 (0.11, 2.30) | 53/162 | 1.34 (0.15, 11.76) |
| PFHxS ^c^ | |  | 162/107 | 0.49 (0.11, 2.30) | 53/162 | 1.34 (0.15, 11.76) |
| PFHpS | |  | 162/108 | 0.12 (0.00, 48.89) | 53/162 | 1.24 (0.00, 1159.32) |
| PFOA | |  | 162/108 | 1.11 (0.37, 3.32) | 53/162 | 0.90 (0.29, 2.80) |
| PFOS | |  | 162/108 | 0.96 (0.84, 1.10) | 53/162 | 1.08 (0.94, 1.24) |
| PFNA | |  | 162/108 | 1.00 (0.20, 5.09) | 53/162 | 1.48 (0.32, 6.82) |
| PFDA | |  | 162/108 | 0.40 (0.02, 8.35) | 53/162 | 1.38 (0.03, 63.03) |
| PFUdA | |  | 162/108 | 0.14 (0.00, 22.91) | 53/162 | 0.27 (0.00, 857.47) |

^a^ Adjusted for age, sex, kidney disease, other chronic disease, national origin, place of testing, and days between blood sampling and diagnosis. Individuals who had blood sampled more than one week before diagnosis, individuals included at Odense University hospital, and individuals who had blood sampled after the first hospitalization day were excluded
^b^ PFBS analyses were conducted for samples from Copenhagen area only

^c^ PFHxS >10 ng/mL excluded
